# An Oral Biodegradable Spiny Milli-Ball Robot for Targeted Intestinal Delivery of Biologics with Delayed Onset and Sustained Action

**DOI:** 10.34133/cbsystems.0716

**Published:** 2026-10-16

**Authors:** Rong Tan, Xiong Yang, Yajing Shen

**Affiliations:** ^1^ Guangdong Provincial Key Laboratory of Robotics and Intelligent System, Shenzhen Institutes of Advanced Technology, Chinese Academy of Sciences, Shenzhen 518055, China.; ^2^ The Department of Electronic and Computer Engineering, Hong Kong University of Science and Technology, Kowloon, Hong Kong 999077, China.; ^3^ Research Center on Smart Manufacturing, Hong Kong University of Science and Technology, Kowloon, Hong Kong 999077, China.

## Abstract

Oral delivery of biologics offers a noninvasive alternative to repeated injections for treating intestinal and systemic diseases, yet it remains challenged by poor gastric protection, limited intestinal targeting, and uncontrolled payload release timing. Conventional passive delivery systems fail to coordinate targeted localization with delayed onset and sustained action, while most ingestible robotic systems rely on non-degradable materials and lack integrated biologic delivery functions. Here, we present an oral biodegradable spiny milli-ball robot for targeted intestinal delivery of biologics with temporally regulated release. The spiny milli-ball robot integrates a magnetic actuation skeleton for controllable locomotion and precise positioning, a core–shell microneedle array for protected payload loading and mucosal penetration, and pH-responsive biodegradable materials for gastric shielding and intestinal activation. Fabricated via 3D-printing-assisted micromolding, the robot exhibits robust mechanical properties for tissue insertion and adaptive locomotion in complex intestinal environments. In vitro characterizations confirm pH-triggered delayed release, negligible gastric leakage, tunable payload capacity, and excellent biocompatibility. In vivo porcine studies validate magnetic-guided intestinal targeting, effective mucosal insertion, and delayed-onset sustained insulin delivery with reduced early-phase exposure compared to subcutaneous injection. This work establishes a biodegradable ingestible robotic platform that unites active intestinal targeting, tissue engagement, and programmed payload release, addressing critical unmet needs for oral biologic delivery.

## Introduction

Oral delivery of biologics could provide a noninvasive alternative to repeated injections for the treatment of intestinal and systemic diseases [1–4]. Yet, effective oral delivery remains difficult because biologic payloads must survive the harsh gastric environment, reach the target intestinal region, and establish productive contact with the local mucosa before meaningful therapeutic action can occur [5–8]. For biologics intended to act after intestinal arrival, therapeutic performance depends not only on whether the payload reaches the intestine but also on where and when exposure begins [9–11]. In this context, intestinal targeting and temporal regulation of onset are closely linked requirements for effective oral biologic delivery, particularly for systems intended to achieve localized administration rather than nonspecific release along the gastrointestinal tract.

Most existing oral delivery systems, including enteric formulations [12], hydrogels [13], and micro/nanocarriers [14,15], are designed around passive gastrointestinal transit followed by environmentally triggered release. These strategies can improve gastric protection and intestinal access, but they generally offer limited control over post-arrival localization, mucosal engagement, and the onset of therapeutic action [16–18]. In many cases, payload exposure begins as soon as local conditions permit swelling, dissolution, or disassembly, regardless of whether the carrier has reached an optimal intestinal site or established robust contact with the mucosa [19,20]. Such premature exposure can reduce local delivery efficiency, weaken the benefit of targeted administration, and shorten the duration of therapeutic effect [21], especially when therapeutic performance depends on maintaining sufficient payload availability after stable contact with the target intestinal region has been established. There therefore remains a need for oral delivery systems that can better coordinate intestinal targeting with delayed onset and sustained therapeutic action.

Ingestible devices and gastrointestinal robotic systems offer a potential route to address this limitation because they can introduce active functions that are largely inaccessible to conventional carriers [22–24], including locomotion, site-specific positioning, retention, and mechanical interfacing with tissue [25,26]. These capabilities create opportunities not only to improve intestinal localization but also to influence when and where payload exposure occurs after oral administration. Active positioning may help the carrier reach desirable intestinal sites more reliably, whereas tissue-interfacing structures may enhance local contact with the mucosa and thereby improve delivery after arrival [11,27]. However, many reported ingestible robotic systems rely on non-degradable components, are designed primarily for sensing or mechanical intervention rather than drug delivery, or do not integrate active targeting with physiologically triggered release [28,29]. A biodegradable robotic system that combines intestinal targeting, tissue engagement, and temporally regulated biologic delivery therefore remains lacking.

Here, we report an oral biodegradable spiny milli-ball robot (SMB-bot) for targeted intestinal delivery of biologics with delayed onset and sustained action. The platform combines protected transit through the upper gastrointestinal tract, pH-responsive activation in the intestinal environment, magnetic actuation for targeted positioning, and a spiny tissue-interfacing architecture that enhances localized engagement with the intestinal mucosa. By integrating these features within a single biodegradable system, the platform is designed to move beyond passive intestinal release and instead support a staged delivery process after oral administration. The resulting delivery profile is characterized by a short but distinct delay in onset rather than an immediate burst-like response after administration. We further present the design and fabrication of the robot, characterize its locomotion and tissue-interfacing behavior, examine its activation and release properties, and evaluate its targeted intestinal delivery performance in vivo. Together, these findings establish a biodegradable ingestible robotic strategy for intestinal biologic delivery that links targeted localization with temporally regulated payload exposure.

## Methods

### Fabrication of the SMB-bot

The SMB-bot was fabricated using a 3D-printing-assisted micromolding process (Fig. S1). First, a resin mold with the designed microneedle geometry was prepared by 3D printing. A poly(dimethylsiloxane) (PDMS) negative mold was then replicated by casting and curing PDMS on the resin template. Using the obtained PDMS mold, the SMB-bot was assembled through a layer-by-layer casting process. Specifically, a Eudragit L100-55 (EL100-55) compound solution, consisting of EL100-55, calcium carbonate, and sodium citrate, was first cast into the mold to form the hollow shell structure. Drug or active-pharmaceutical-ingredient powder was then manually loaded into each microwell. Subsequently, a 20% hyaluronic acid methacrylate solution was cast and ultraviolet cured to form the backing layer, followed by casting of a 20% *w*/*v* poly(vinyl alcohol) (PVA) solution as the binder layer. Finally, a tissue adhesive (3M)/PVA mixture was added to seal the hemispherical shell and embed the magnetic skeleton, yielding the final SMB-bot.

### Mechanical characterization of the microneedles

The hardness of the microneedle material was measured using a nanoindenter (Hysitron TI 980; Bruker, USA), with unsynthesized EL100-55 serving as the control. Nanoindentation was performed under load-control mode at a loading/unloading rate of 0.2 mN/s up to a peak load of 1 mN, and the indentation hardness was extracted from the recorded load–displacement curves. The mechanical strength of the SMB-bot microneedles was further evaluated by compression testing using a universal testing machine (Instron 5943; Instron, USA). A vertical compressive load was applied to the microneedles at a constant crosshead speed of 4 mm/min until deformation or failure, and the corresponding force–displacement curves were recorded to determine the maximum compressive force.

### In vitro SMB-bot biodegradation test

A chamber containing agar (Sigma-Aldrich, 1 wt.%, 1 mL) was used as an artificial tissue model, and a parafilm layer was placed on top as a protective barrier. The SMB-bot was inserted through the parafilm into the agar and then immersed in simulated intestinal fluid. Fluorescein isothiocyanate (FITC)–insulin and a red fluorescent marker were incorporated into the SMB-bot to visualize the degradation and release process. At predetermined time points, the structural and fluorescence changes of the SMB-bot were recorded using fluorescence microscopy and optical imaging.

### Drug-loading quantification of SMB-bots with different microneedle lengths

To investigate the effect of microneedle length on drug-loading capacity, SMB-bots with different microneedle lengths were fabricated, and insulin powder was manually packed into the hollow microneedle cavities. The loaded insulin in each robot was then extracted in 37 °C phosphate-buffered saline (PBS) and quantified using a human insulin enzyme-linked immunosorbent assay (ELISA) kit, and the results were expressed as insulin units (U) per robot.

### In vitro insulin release test of the SMB-bot

Insulin-loaded SMB-bots were first incubated in simulated gastric fluid (pH 1.2) at 37 °C under gentle shaking for 30 min and then transferred to simulated intestinal fluid (pH 6.8) to trigger pH-responsive release. At predetermined time points, aliquots of the release medium were collected and replaced with an equal volume of fresh medium. The insulin concentration in the collected samples was quantified using a human insulin ELISA kit according to the manufacturer’s instructions. The cumulative insulin release was calculated relative to the total insulin loading in each SMB-bot. All experiments were performed in triplicate.

### In vitro cytotoxicity of the SMB-bot

L929 fibroblast cells were cultured in Dulbecco’s modified Eagle’s medium supplemented with 10% fetal bovine serum and 1% penicillin–streptomycin at 37 °C in a humidified 5% CO_2_ incubator. For the cytotoxicity assay, 200 μL of cell suspension (1.0 × 10^4^ cells/mL) was seeded into each well of a 96-well plate and incubated for 24 h. One SMB-bot was dissolved in 1 mL PBS (pH 7.0 to 7.2) at 37 °C for 24 h to prepare the extract solution, which was then diluted to final concentrations of 1.56, 3.125, 6.25, 12.5, 25, 50, and 100 mg/mL. The cells were treated with the corresponding extracts for 24 h, followed by incubation with 3-(4,5-dimethylthiazol-2-yl)-2,5-diphenyltetrazolium bromide (MTT) solution (5 mg/mL) for 4 h. After removing the medium, 200 μL dimethyl sulfoxide was added to dissolve the formazan crystals, and the absorbance at 570 nm was measured using a microplate reader. Three independent experiments were performed, with 6 replicates per group.

### In vivo experiment

All in vivo experiments were performed at Shenzhen Lingfu Topu Biotechnology Co., Ltd. (Approval No. TOPGMIACUC-2024-0380) in accordance with institutional animal welfare and ethical guidelines. Four healthy Bama miniature pigs (female, 6 to 9 mo old; body weight 15 to 35 kg) were used in this study. Animals were acclimated under standard housing conditions before experimentation and fasted on the day of the procedure. General anesthesia was induced prior to all procedures, and the animals were maintained under anesthesia throughout the experiments.

Of the 4 pigs, one was assigned to evaluate the in vivo locomotion and insertion performance of the SMB-bot. To establish an intestinal protrusion model for insertion validation, laparotomy was first performed to expose the target intestinal segment. A 0.3% *w*/*v* low-temperature gelatin solution (low-gelling-temperature agarose, A9414; Sigma-Aldrich, USA) was then injected into the submucosal layer to create an artificial intestinal protrusion, after which the intestine and abdominal wall were sutured closed. The SMB-bot was subsequently delivered into the gastrointestinal tract using a combined endoscope-and-tube-assisted approach. Under endoscopic guidance, the delivery tube was advanced to the target site, and the SMB-bot was introduced through the tube. External magnetic actuation was then applied to guide the locomotion of the robot and promote its insertion into the gelatin-induced protrusion model. The movement and insertion behaviors of the SMB-bot were monitored and recorded by endoscopy.

The remaining 3 pigs were used for in vivo blood glucose studies. In these animals, the SMB-bot was directly delivered to the target intestinal site using the same endoscope-and-tube-assisted approach, followed by magnetic actuation to achieve insertion. Blood samples were collected at predetermined time points, and blood glucose levels were measured using a glucometer. Serum insulin concentrations were further quantified using a human insulin ELISA kit according to the manufacturer’s instructions. The area under the curve was calculated from the blood glucose and serum insulin profiles for quantitative analysis.

## Results

### Design and characterization of the SMB-bot

To integrate locomotion, payload carrying, and intestinal mucosa penetration within a single oral delivery platform, we designed a biodegradable SMB-bot composed of a magnetic skeleton for actuation and a core–shell microneedle array for protected payload loading and mucosal insertion (Fig. 1A). The magnetic skeleton (gelatin with iron particles [30]) served as the actuation unit of the robot, providing magnetic responsiveness for rolling locomotion and targeted positioning under an external magnetic field, whereas the surrounding microneedle array enabled protected payload loading and tissue penetration. The overall milli-ball geometry provided a compact form suitable for oral administration, and the dimensions of the SMB-bot were selected to allow encapsulation within a commercially available size 00 capsule, thereby enabling delivery by capsule swallowing (Fig. S2). In addition, the spiny microneedles distributed over the robot surface created a multidirectional tissue-interfacing architecture for local contact and insertion after intestinal arrival. As shown in Fig. 1B, each microneedle contained a core–shell microstructure generated by a sandwich-layer covering strategy in which a drug- or active-pharmaceutical-ingredient-containing core was enclosed by a cap layer and a shell layer. These components were formed from biodegradable polymers, including EL100-55, hyaluronic acid methacrylate, and PVA, which together enabled protected loading of the payload before release (Fig. 1B). After oral administration in a commercial capsule, the SMB-bot was transported through the upper gastrointestinal tract and reached the anterior segment of the small intestine, where magnetic actuation enabled targeted positioning and the spiny microneedles facilitated local mucosal insertion (Fig. 1C). Following activation of the outer structure, the loaded drug could be released from the microneedle core and subsequently absorbed through the local capillary network. This integrated design established the structural basis for the magnetic locomotion, tissue engagement, and payload release behaviors characterized in the following sections.

**Fig. 1.**
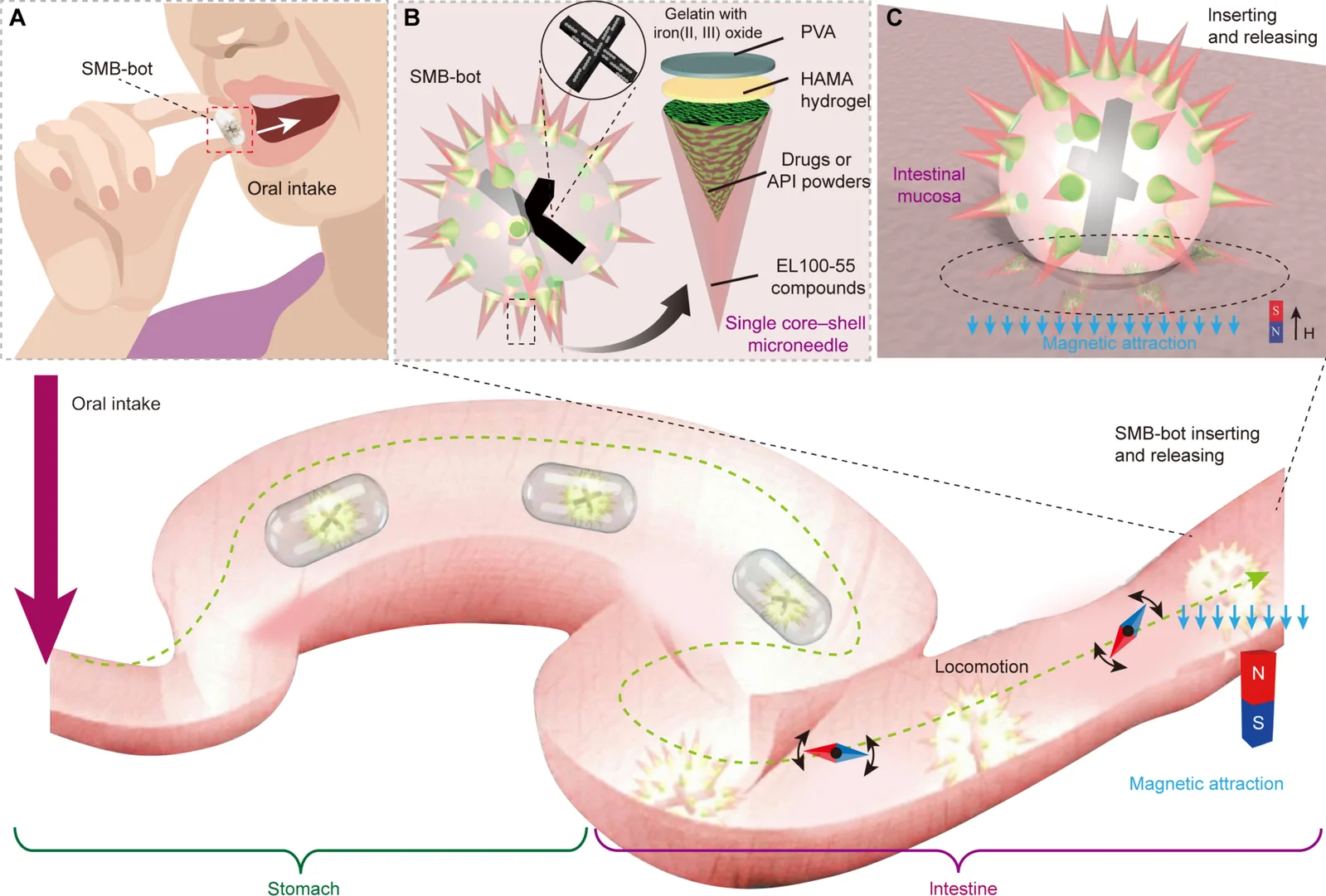
Schematic illustration of the spiny milli-ball robot (SMB-bot) for oral delivery, intestinal locomotion, and magnetically assisted microneedle insertion. (A) Concept of oral administration of the SMB-bot in capsule form. (B) Structural design of the SMB-bot and the configuration of the single core–shell microneedle, comprising a Eudragit L100-55 (EL100-55) compound shell, drug or active pharmaceutical ingredient (API) powder payload, a hyaluronic acid methacrylate (HAMA) hydrogel layer, and a poly(vinyl alcohol) (PVA) sealing layer containing gelatin with iron oxide for magnetic responsiveness. (C) Working principle of the SMB-bot under an external magnetic field, where magnetic attraction enables the robot to approach the intestinal mucosa, followed by microneedle insertion and payload release. The lower panel illustrates the overall operation route of the SMB-bot after oral intake, including gastric transit, intestinal locomotion, and targeted magnetic actuation for local insertion and drug release in the intestine.

The SMB-bot was fabricated through a combined 3D-printing-assisted micromolding strategy (see the “Methods” section), whereas the detailed fabrication procedure is described in the “Methods” section. Briefly, a 3D-printed resin template was used to define the overall geometry and microneedle architecture of the robot, from which a PDMS mold was replicated for subsequent multistep casting and assembly. This approach enabled the formation of a hemispherical robot body with a spiny microneedle array and supported integration of the magnetic skeleton, drug-containing core, and biodegradable shell layers within a single construct.

To characterize the resulting morphology of the fabricated SMB-bot, we first examined its surface architecture by scanning electron microscopy. As shown in Fig. 2A-i, the robot exhibited a compact spiny milli-ball geometry with an ordered array of microneedles distributed over the hemispherical surface. The overall diameter of the SMB-bot was 6.5 mm, which is compatible with encapsulation in a commercially available size 00 capsule for oral administration. Each microneedle displayed a tapered profile with a height of ~600 μm, a base diameter of ~300 μm, and a tip angle of ~22.7° (Fig. 2A-ii). The resultant robot was found to have an orderly array of tips and a tiny size. Besides, by staining the 3 components with different fluorescent dyes, the core–shell structure of the robot could be clearly characterized. To assist the hollow structure characterization, rhodamine was put into the shell solution, and the FITC–insulin was pressed into the microneedle’s hole (Fig. 2A-iii). Through peeling off the shell of the microneedle, the insulin-lined hills can be clearly identified as shown in Fig. 2B-ii and B-iii. Meanwhile, from the scanning electron microscopy image, we can clearly judge that the bonding part of the binder is very tight. The thickness of the bonding surface is about 180 μm (Fig. 2B-iv).

**Fig. 2.**
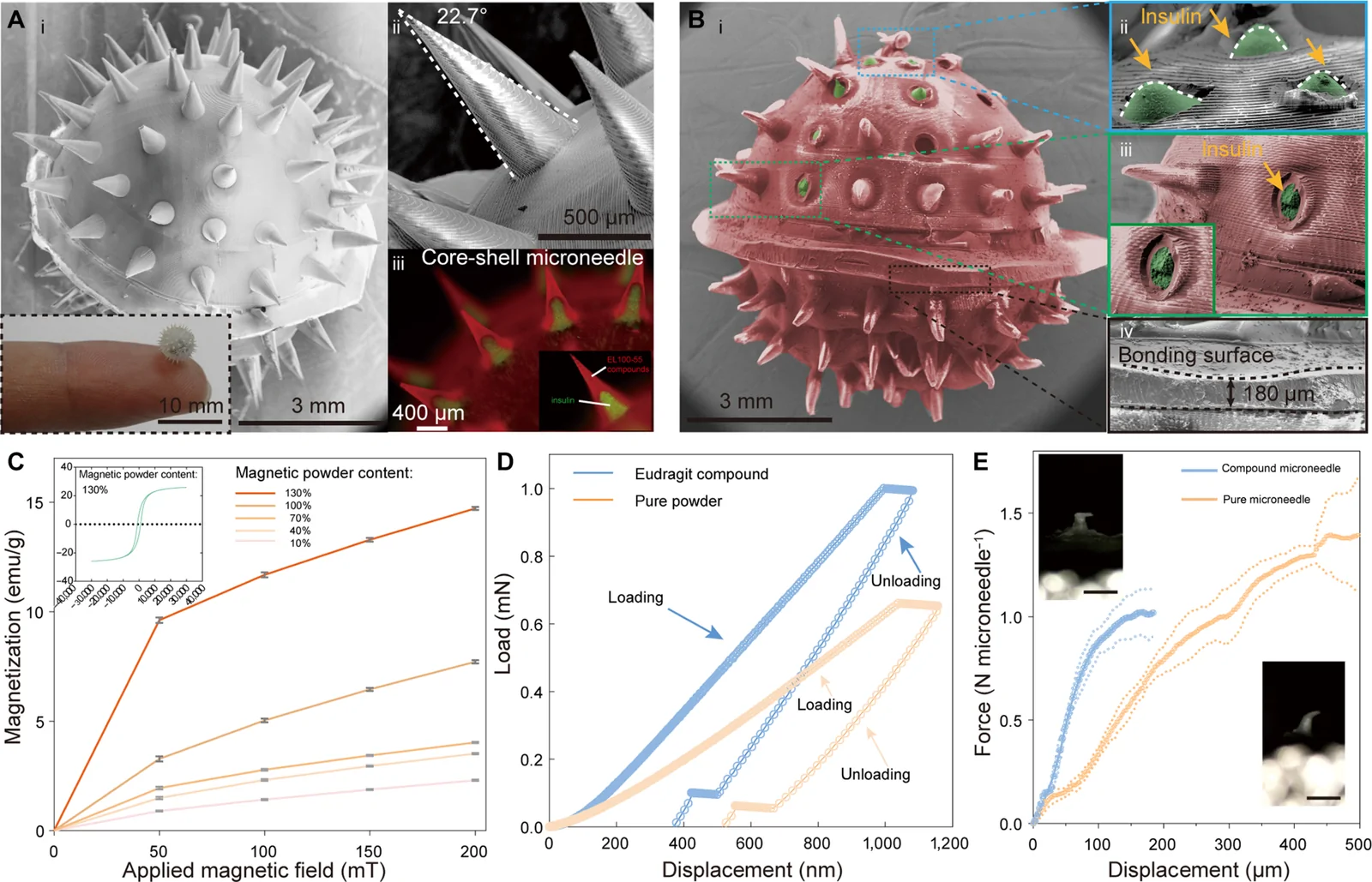
The structure and functional characterization of the SMB-bot. (A) The morphology characterization of the SMB-bot’s body. The fluorescence image clearly characterizes the core–shell structure of the SMB-bot. (B) The morphology characterization of core–shell microneedle. (C) The magnetization of the magnetic actuating skeleton (gelatin solution concentration 15% and magnetic microparticle content 130%). As applied magnetic field strength increases from 0 to 200 mT, the measured magnetization increases from 0 to 14.9 emu/g. (D) The load–displacement curve of test points on the composite microneedle’s material measured using a nanoindenter. The average indentation hardness of the microneedle is ~37 MPa, which is enough to puncture various tissues and epidermis. (E) Analysis of the compressive forces of the microneedle robots. Changes of the compressive force over time (synthesized/unsynthesized).

To guarantee the robustness of the SMB-bot, the mechanical performance of the core–shell microneedle structure and the magnetic actuating skeleton will also be evaluated. From the perspective of robot locomotion, the magnetic strength should affect the reliability of the SMB-bot during locomotion. Therefore, we will test the magnetic actuating skeleton’s magnetic strength under the different ratios of gelatin and Fe_3_O_4_ nanoparticles that meet insert mechanical strength and motion flexibility requirements. In addition, we will evaluate the mechanical performance of the core–shell microneedle structure, including indentation hardness and penetration force. The magnetic actuating skeleton’s magnetic moment density is characterized by different magnetic field strengths ranging from 0 to 200 mT. As the curve shows in Fig. 2C, the magnetization of the skeleton increases with the growth of the magnetic powder’s content, which was experimentally measured using a vibrating sample magnetometer. When the content is 130%, the magnetization of the skeleton’s leg rises from 0 to 14.9 emu/g with increased applied magnetic field strength. This positive correlation between magnetization and magnetic field strength makes it possible for the stable and controllable actuation of the SMB-bot under a magnetic field.

To ensure that the SMB-bot could be inserted into the mucosa of the small intestine, we needed to characterize the materials and morphology of the microneedle. We first adopt the nanoindenter to measure the hardness of the microneedle’s materials (Fig. 2D). Here, we set an unsynthesized EL100-55 as a blank control group. Compared with the composite Eudragit we choose, the average indentation hardness of the composite is ~37 MPa, which is higher than the unsynthesized hardness of ~30 MPa. Meanwhile, the average indentation hardness of the composite is higher than various tissues and epidermis [31], meaning it is hard enough for puncturing. The SMB-bots should be strong enough to penetrate tissues under compression without fracturing. For this purpose, compressive-force tests were conducted to evaluate the mechanical strength of these SMB-bots (Fig. 2E). Results show that the maximum compressive force of the synthesized microneedle is higher than the unsynthesized one. The SMB-bots with synthesized shells could tolerate about 1.25 N per microneedle, indicating a successful tissue penetration.

### Magnetic actuation and tissue-interfacing behavior enable targeted intestinal engagement

The magnetic actuation skeleton and spiny microneedle architecture of the SMB-bot collectively endow the system with controllable locomotion, adaptive navigation, and reliable mucosal penetration, which are essential for targeted intestinal engagement after oral administration. The magnetic actuation skeleton (15 wt.% gelatin and 130 wt.% Fe_3_O_4_ nanoparticles) serves as the core actuation component, with a saturation magnetization of 14.9 emu/g at 200 mT, providing sufficient magnetic responsiveness for in vivo manipulation. It was noted that increasing Fe_3_O_4_ nanoparticle content improves magnetic responsiveness; however, excessive nanoparticle loading increases stiffness and may reduce structural integrity and adaptability during locomotion. Meanwhile, gelatin concentration affects both mechanical stability and flexibility. Therefore, 15 wt.% gelatin with 130 wt.% Fe_3_O_4_ nanoparticles was selected because this composition provided sufficient magnetic response while maintaining the mechanical flexibility required for rolling locomotion and tissue interaction. Under an external magnetic field (generated by a permanent magnet mounted on a Universal Robots robotic arm platform), the magnetic skeleton simultaneously experiences magnetic torque and magnetic gradient pulling force, which jointly provide stable driving force for directional rolling locomotion and precise target positioning (Fig. 3A). Mechanical analysis shows that the magnetic actuation system can effectively convert external magnetic field energy into mechanical motion displacement, and the dynamic equilibrium equation satisfies $F_{m}-F_{f}-F_{\eta }=m_{\mathrm{bot}}a$, where *F*_m_ is the magnetic pulling force or translational movement derived from the magnetic field gradient, *F*_f_ is the sliding friction force, *F_η_* is the viscous resistance of the intestinal mucus, *m*_bot_ is the total mass of the SMB-bot, and *a* is the acceleration of the robot, ensuring that the SMB-bot can stably overcome the resistance of the intestinal mucus and maintain directional linear motion in the complex intestinal lumen environment (Fig. 3B).

**Fig. 3.**
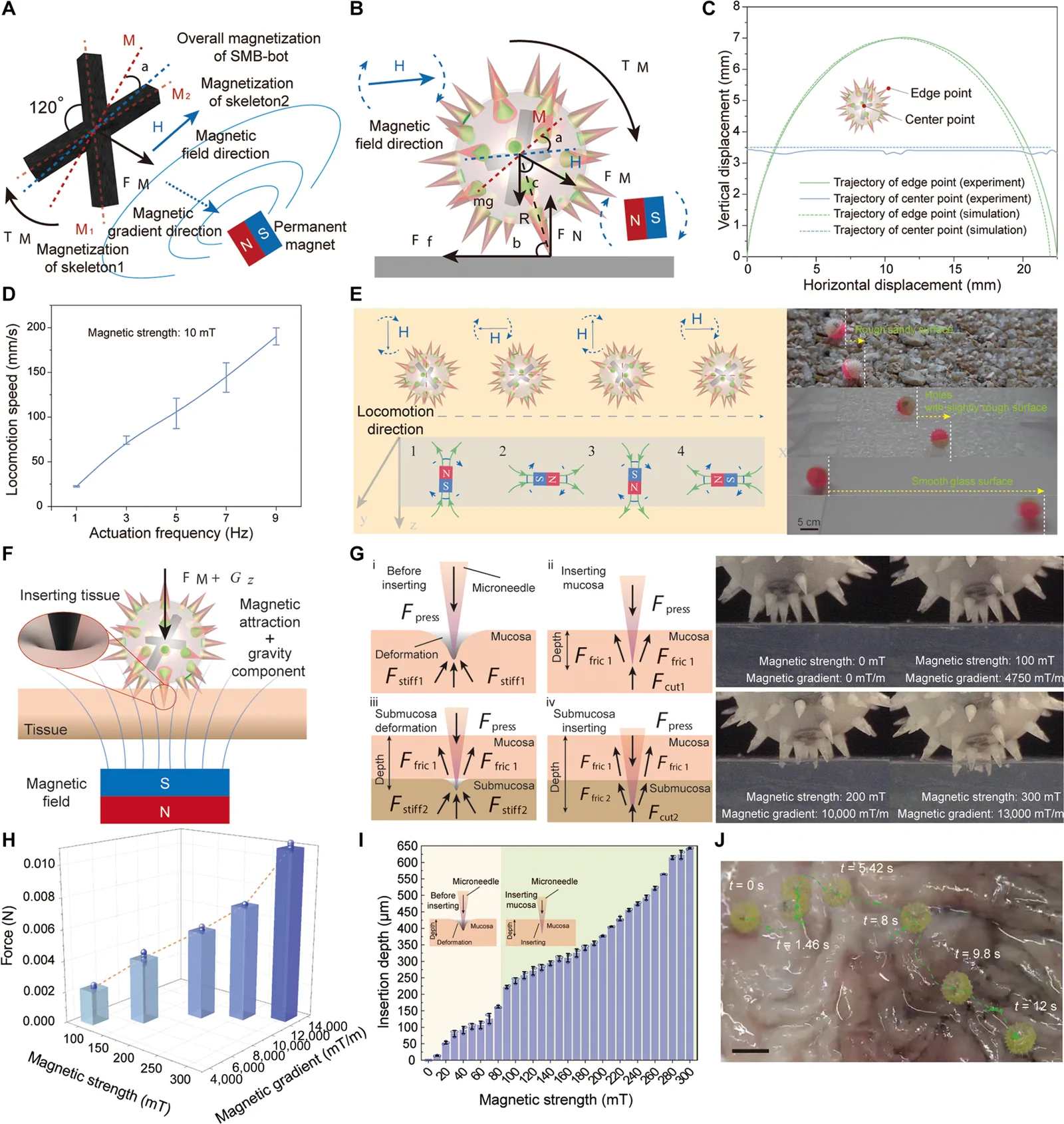
Actuation and penetration control of the SMB-bot. (A) The magnetic actuation skeleton will experience both torque and pulling force under the action of the magnetic field. (B) Mechanical analyses of the SMB-bot under the magnetic field. (C) Comparison between the theoretical model and real motion trajectory. The actual motion experiment is consistent with the simulation results of the model. (D) The velocity of the SMB-bot under the magnetic field with different frequencies. (E) The adaptive motion of the SMB-bot on different rough surfaces (rough sandy surface, holes with slightly rough surface as well as smooth glass surface). (F) Schematic diagram of the SMB-bot puncture under the combined action of magnetic attraction and gravity. (G) Mechanical analyses of the microneedle during the agar insertion process. Puncture testing of the SMB-bot on tissue-like agar under the magnetic field with different strengths and gradients. (H) Changes of the force per microneedle as the microneedle robot gradually penetrates into the agar. (I) Changes in the magnetic force generated by the approaching magnet. The *x*-axis means the distance between the fixed SMB-bot’s microneedle and the mobile magnet. (J) The versatile locomotion performance of the SMB-bot. The target drug delivery of the SMB-bot in a real pig stomach. The pig stomach surface is covered with a mucus layer (pH 5 and viscosity 400 mPa·s). Scale bar: 10 mm.

To quantitatively verify the accuracy of magnetic actuation control, a kinematic model of the rolling motion of the SMB-bot was established (Text S1), and the theoretical motion trajectory was fitted and compared with the actual experimental trajectory. The results show that the theoretical simulation trajectory is highly consistent with the actual motion path of the robot, and the trajectory deviation is less than 5%, which fully confirms the high precision and reliability of the magnetic actuation control strategy (Fig. 3C). The locomotion velocity of the SMB-bot can be flexibly regulated by adjusting the frequency of the external magnetic field. In the frequency range of 1 to 9 Hz, the robot velocity shows a good linear positive correlation with the magnetic field frequency, that is, $v=\frac{D}{2}\cdot \omega =\frac{D}{2}\cdot \frac{\left|\tau \right|}{I_{\mathrm{rot}}}$, where *v* is the linear velocity of the rolling robot, *D* is the robot diameter, *w* is the angular velocity, *τ* is the magnetic torque governing rotational locomotion, and *I*_rot_ is the rotational inertia. When the magnetic field amplitude is fixed, the velocity increases from 25 to 180 mm/s as the frequency increases, demonstrating excellent motion controllability and speed adjustability (Fig. 3D). In addition, the adaptive locomotion ability of the SMB-bot was tested on 3 kinds of surfaces that simulate the intestinal microenvironment: rough sandy surface, slightly porous rough surface, and smooth glass surface. The results show that the robot can maintain stable rolling motion without jamming or slipping on all the above surfaces, indicating that it has strong environmental adaptability and can cope with the heterogeneous and slippery characteristics of the intestinal tract wall (Fig. 3E).

To further verify the in vivo application feasibility of the SMB-bot, versatile locomotion and mucosal penetration tests were carried out on the isolated porcine stomach model. The experimental environment was set to be consistent with the physiological state: The surface was covered with a thick mucus layer. The results show that the SMB-bot can still maintain stable rolling locomotion under the drag of high-viscosity mucus, and the microneedle can successfully penetrate the mucosal layer, achieving noninvasive and stable targeted engagement (Fig. 3J). These results systematically confirm that the magnetic actuation and tissue-interfacing characteristics of the SMB-bot can realize reliable targeted localization and stable mucosal attachment in the intestinal tract, which provides a solid mechanical and functional foundation for the subsequent delayed onset and sustained release of biological agents.

For efficient and stable intestinal mucosal engagement, the SMB-bot realizes controllable tissue penetration under the synergistic drive of vertical magnetic attraction and gravity component, and the total penetration force satisfies $F_{\text{pen}}=F_{\text{mag},⊥}+\text{mg}\text{cos}\theta$ (Fig. 3F), where *F*_pen_ is the total penetration force, *F*_mag,**⊥**_ is the normal magnetic force, *m* is the mass of the SMB-bot, *g* = 9.8 m/s^2^, and *θ* is the inclination angle of the robot relative to the tissue surface. Mechanical analysis and penetration tests on mucosa-mimicking agar substrate show that the core–shell microneedle structure has an indentation hardness and a single needle can withstand a maximum compressive force, which is sufficient to penetrate the mucosal matrix without structural fracture (Fig. 3G and Video S1). In this study, the agar substrate was used as a simplified tissue phantom to evaluate the penetration capability of the SMB-bot microneedles under controlled and repeatable conditions. The agar phantom was prepared to provide a hydrated, soft, and homogeneous matrix for evaluating the mechanical interaction between the microneedles and a tissue-like environment. With the gradual insertion of the microneedle into the agar, the penetration force per microneedle increases steadily from 2 to 10 mN, and the robot can stay stably at the target site without detachment after penetration (Fig. 3H). Meanwhile, the vertical magnetic force generated by the external magnet decreases with the increase of the distance between the magnet and the robot, which can realize the precise regulation of penetration depth and avoid excessive pressure causing mucosal damage (Fig. 3I and Video S2).

### In vitro pH-triggered release, payload loading, and biocompatibility of the SMB-bot

To evaluate the pH-triggered release behavior, payload loading capability, and in vitro biocompatibility of the SMB-bot, we next examined the drug delivery performance of its core–shell structure under tissue-mimicking and gastrointestinal relevant in vitro conditions (Fig. 4A). The SMB-bot was constructed with a multilayer microneedle architecture in which the payload was confined within the inner compartment and protected by the surrounding shell and cap layers. After insertion into a mucosa-mimicking agar substrate, the outer structure was intended to remain stable in the acidic gastric environment and then gradually dissolve upon exposure to intestinal pH (pH > 5.5, 37 °C), thereby allowing subsequent payload release. This schematic outlines the working principle of the SMB-bot for protected oral delivery and intestinally triggered release.

**Fig. 4.**
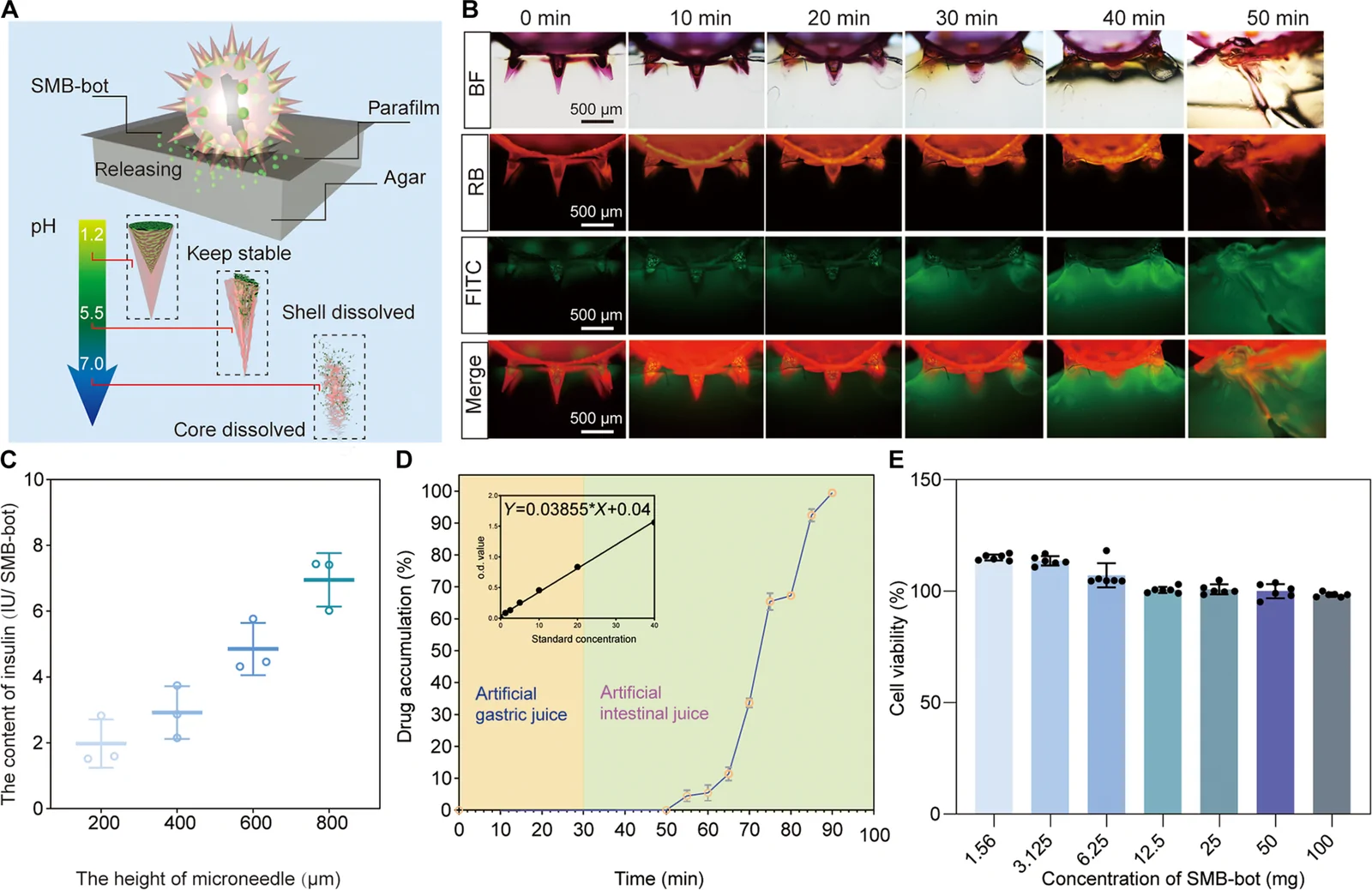
In vitro payload loading, pH-triggered release, and biocompatibility of the SMB-bot. (A) Schematic illustration of the pH-responsive behavior of the SMB-bot, showing structural stability under acidic conditions and activation in the intestinal environment. (B) Representative fluorescence images showing time-dependent release from the SMB-bot microneedle structure in an alkaline environment, 37 °C. FITC, fluorescein isothiocyanate; RB, Rhodamine B; BF, bright field. (C) Payload loading capacity of SMB-bots with different microneedle heights. (D) Cumulative release profile of the payload in artificial gastric juice and artificial intestinal juice, showing limited release under gastric conditions and activated release in intestinal conditions. Inset, linear fitting of the initial intestinal-phase release profile. o.d., optical density. (E) Cell viability after incubation with different concentrations of SMB-bots, showing good in vitro biocompatibility.

To verify this process experimentally, we monitored the structural evolution of the microneedles and the diffusion of the loaded payload by fluorescence imaging under the corresponding in vitro alkaline conditions (Fig. 4B). For visualization, the outer shell was labeled with a red fluorescent dye, while FITC powder was loaded into the microneedles as a model fluorescent payload. At the initial stage, the microneedles retained their overall morphology, and the red fluorescent shell remained clearly localized around the payload-containing region. After 10 to 20 min under alkaline conditions (pH > 5.5, 37 °C), the red fluorescence began to weaken, while the green signal gradually diffused into the surrounding medium, indicating the onset of shell dissolution and payload release. This trend became more pronounced after 30 min, when attenuation of the shell signal was accompanied by further outward spreading of the FITC-labeled payload. With continued incubation to 40 min, the microneedle contours became increasingly indistinct, suggesting substantial disintegration of the outer structure. By 50 min, the original microneedle morphology had largely disappeared, and the green fluorescence had spread extensively into the surrounding region. The fluorescence images therefore verify a time-dependent release process, in which progressive shell dissolution is accompanied by continuous outward diffusion of the payload. These observations show that the SMB-bot undergoes time-dependent structural disintegration together with payload release in the tissue-mimicking environment.

We further quantified the loading capacity of the SMB-bot using insulin as a model drug (Fig. 4C). To determine the total amount of loaded insulin, SMB-bots with different microneedle heights were dissolved in alkaline PBS at 37 °C, and the insulin content was quantified by ELISA kit (see experimental procedures). The insulin loading amount increased with microneedle height, rising from approximately 2 IU to nearly 7 IU per robot as the microneedle height increased from 200 to 800 μm. This result indicates that the loading capacity of the SMB-bot can be tuned through geometric design of the microneedles. The higher loading amount in taller microneedles is consistent with the larger internal cavity volume available for drug encapsulation.

We next quantified release under sequential artificial gastric and intestinal conditions (Fig. 4D and Fig. S3). In artificial gastric juice, almost no release was detected, indicating effective protection of the payload under acidic conditions. After transfer to artificial intestinal juice, the release curve remained nearly flat for the first 20 min before rising sharply. Combined with the fluorescence images in Fig. 4B, this lag phase is consistent with dissolution of the outer shell before the payload becomes exposed. Once the shell had dissolved, release proceeded rapidly and approached completion. These data indicate a 2-step release process in which shell removal precedes payload liberation.

To quantitatively evaluate the release profile of the SMB-bot, we carried out in vitro dissolution tests under sequential gastric and intestinal conditions (see experimental procedures). In the gastrointestinal tract, the luminal pH changes rapidly from the strongly acidic stomach to the weakly acidic or neutral small intestine. Correspondingly, a similar pH transition was adopted in the in vitro characterization to assess whether the SMB-bot could protect the payload during gastric transit and release it after intestinal exposure. As shown in Fig. 4D, almost no detectable drug release occurred during the first 30 min in artificial gastric juice, with cumulative release remaining close to 0%, indicating that the encapsulated payload was well protected under acidic conditions. After transfer to artificial intestinal juice, the release curve remained nearly flat for a further ~20 min, reaching only ~4% to 5% at 55 min and ~5% to 6% at 60 min. A clear increase was first observed at 65 min, when cumulative release rose to ~11%, followed by a sharp increase to ~33% at 70 min and ~66% at 75 min. The release continued to increase thereafter, reaching ~68% at 80 min, ~92% to 93% at 85 min, and nearly complete release by 90 min. This delayed rise in the release curve indicates that payload liberation did not occur immediately upon intestinal exposure, but only after completion of an outer-shell dissolution stage. Combined with the fluorescence images in Fig. 4B, the ~20-min lag phase is consistent with the time required for shell removal before the encapsulated payload became exposed. The release behavior therefore supports the delayed-onset and sustained-action design of the SMB-bot for intestinal delivery of biologics.

The in vitro cytotoxicity tests were also implemented to evaluate the biocompatibility of the SMB-bot with a series of concentration gradients (ranging from 1.56 to 100 mg) via MTT assay. Here, multiple groups of MTT assays were independently carried out using a normal cell line (L929 fibroblast cells). As shown in Fig. 4E, the viability of cells treated with SMB-bot extracts remained comparable to that of the control group across the tested concentration range, indicating low cytotoxicity of the SMB-bot (see experimental procedures, Fig. S4).

### In vivo targeted intestinal delivery and functional evaluation of the SMB-bot

The in vivo performance of the SMB-bot was evaluated in a porcine model to determine whether it could achieve targeted intestinal locomotion, magnetic insertion, and delayed drug delivery under physiologically relevant conditions. As illustrated in Fig. 5A, the SMB-bot was designed to move along the intestinal surface under magnetic control and to be further attracted toward a predefined tissue bulge for localized insertion (Video S3). To validate this targeting process in vivo, an artificial intestinal protrusion was first created by submucosal injection of low-temperature gelled gelatin, which acted as a reproducible and endoscopically identifiable landmark to evaluate whether the SMB-bot could be magnetically guided from a distant location to a predefined target and subsequently inserted at that specific site. The endoscopic delivery approach was adopted in the current animal experiments to ensure accurate placement, facilitate visualization, and enable controlled evaluation of targeting and insertion performance. Endoscopic images showed that the SMB-bot could be magnetically guided from a distant location toward the target protrusion and subsequently inserted at the designated site under magnetic actuation (Fig. 5B), demonstrating its ability to achieve controlled locomotion and site-specific engagement in the living intestine. To further verify the insertion capability of the SMB-bot in vivo, histological analysis was performed after magnetic actuation. As shown in Fig. 5C, the microneedles penetrated through the mucosa region, confirming effective insertion of the robot into intestinal tissue in the living porcine intestine. The SMB-bot was designed to achieve sufficient mucosal penetration for stable attachment and local delivery while avoiding penetration into deeper intestinal layers.

**Fig. 5.**
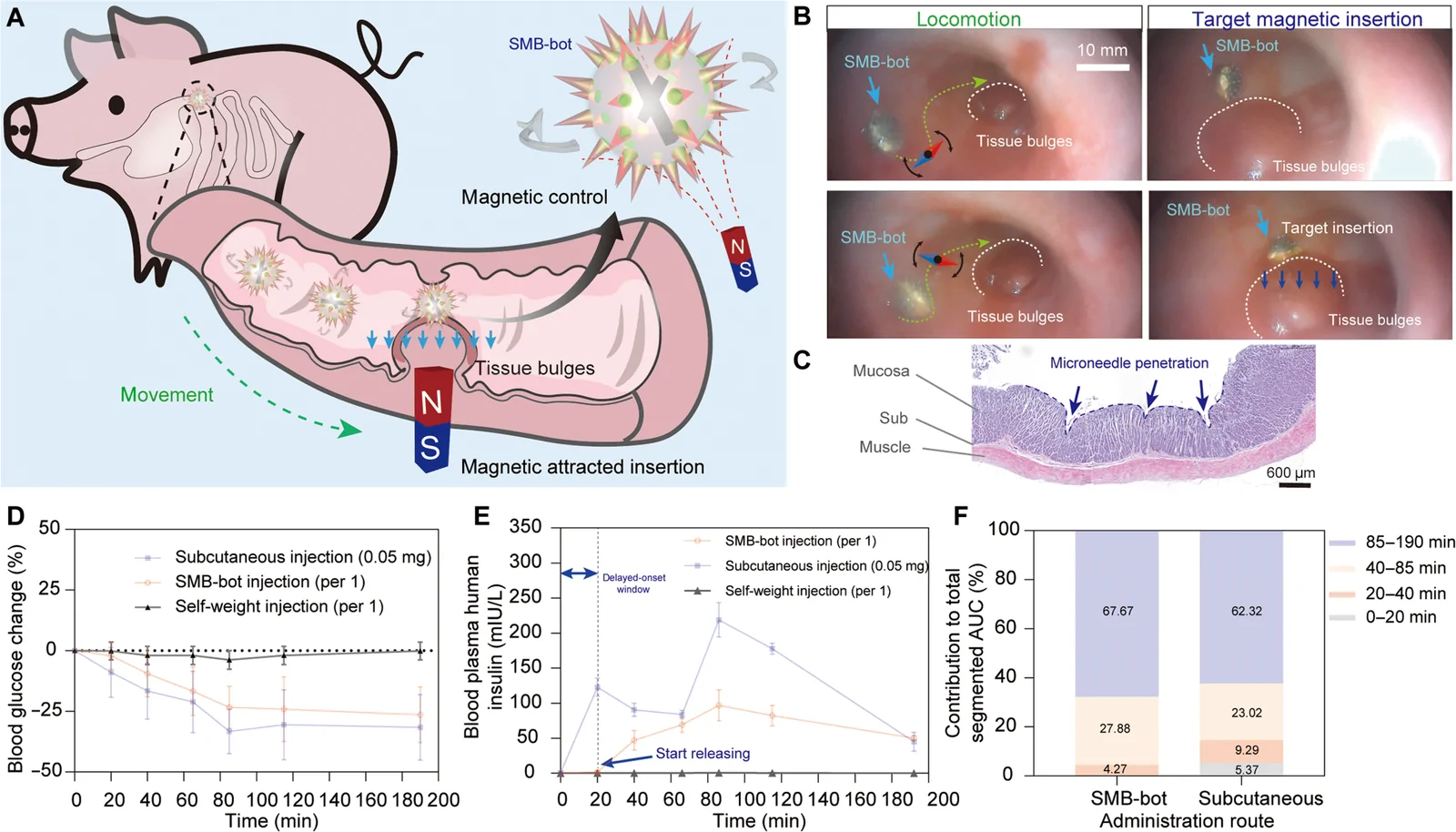
In vivo targeted intestinal delivery and therapeutic evaluation of the SMB-bot. (A) Schematic illustration of in vivo targeted intestinal delivery of the SMB-bot under magnetic guidance in a porcine model. (B) Endoscopic images showing locomotion and magnetically guided insertion of the SMB-bot at the target intestinal site. (C) Histological image showing microneedle penetration into the intestinal mucosa after targeted delivery. (D) Blood glucose responses following SMB-bot delivery, subcutaneous injection, and self-weight injection. (E) Blood plasma human insulin concentration–time profiles following different administration routes, showing a delayed-onset and more moderated early-phase response for SMB-bot delivery. (F) Segmented exposure analysis showing temporal redistribution of insulin exposure after SMB-bot delivery. AUC, area under the curve.

This magnetically enabled insertion was then coupled with insulin delivery to examine the in vivo release behavior of the SMB-bot. Human insulin as model biologics was loaded into the robot and delivered endoscopically into the duodenum (see the “Methods” section), with subcutaneous injection and self-weight placement used as controls. As shown in Fig. 5D, the SMB-bot group produced a progressive reduction in blood glucose, with the glucose decrease expanding from about 10% at 40 min to about 24% at 80 min, and reaching approximately 27% by 190 min. In contrast, subcutaneous injection induced a faster and stronger glucose-lowering effect, with a reduction of about 10% already observed at 20 min and a maximal decrease of approximately 34% at 80 min. By comparison, the self-weight group remained close to baseline throughout the experiment, indicating that passive contact alone was insufficient for effective delivery.

The serum human insulin profiles further supported this interpretation (Fig. 5E). In the SMB-bot group, insulin remained undetectable during the first 20 min and then gradually increased to 48 to 68 mU/L during 40 to 60 min, reaching a peak of about 96 mU/L at 80 min before declining thereafter. By contrast, subcutaneous injection led to immediate systemic exposure, with insulin already reaching about 124 mU/L at 20 min and peaking at approximately 214 mU/L at 80 min. No detectable insulin signal was observed in the self-weight group at any time point. The absence of detectable insulin in the SMB-bot group during the first 20 min, despite the subsequent rise in circulating insulin and the associated glucose reduction, indicates that drug release did not occur immediately upon insertion, but instead required dissolution of the outer shell before payload exposure and absorption.

This delayed-onset behavior was further quantified by segmented area-under-the-curve analysis (Fig. 5F). For the SMB-bot, early-phase exposure was minimal, with essentially no contribution during 0 to 20 min and only 4.27% of the total insulin exposure occurring during 20 to 40 min. In contrast, the corresponding early-phase contribution in the subcutaneous group reached 14.66% in total. The SMB-bot instead showed a greater contribution in later phases, with 27.88% and 67.67% of the total exposure occurring during 40 to 85 min and 85 to 190 min, respectively. These data indicate that the SMB-bot shifted a larger fraction of drug release to later time windows, consistent with a delayed-onset and sustained-action delivery profile in vivo.

Collectively, these results demonstrate that the SMB-bot can be magnetically guided to a predefined intestinal target, effectively inserted into intestinal tissue in vivo, and subsequently achieved temporally programmed delivery of a macromolecular payload.

## Conclusion

We developed a biodegradable SMB-bot that enables oral, targeted, delayed-onset, and sustained intestinal delivery of biologics. By merging magnetic actuation, microneedle-based tissue interfacing, and pH-responsive biodegradation, the SMB-bot overcomes key limitations of passive carriers and non-degradable robotic systems. Previous intestinal delivery systems have primarily focused on 1 or 2 aspects, such as gastric protection, intestinal localization, or mucosal penetration. For example, enteric coatings and micro/nanocarriers can protect payloads from gastric degradation but generally rely on passive gastrointestinal transit and lack active positioning. Ingestible robotic systems provide active manipulation capabilities but often rely on non-degradable materials or focus on sensing, biopsy, or mechanical intervention rather than integrated biologic delivery [2,3,11,16,23,27,29].

In contrast, our robot safely transits the gastric tract, navigates to intestinal targets under external magnetic control, penetrates the mucosa for stable retention, and releases payloads only after shell dissolution, yielding a delayed and sustained delivery profile. In vitro and in vivo results demonstrate reliable locomotion, effective mucosal insertion, minimal gastric release, tunable drug loading, and favorable biocompatibility. During the experiments, the penetration force increased gradually during insertion, reaching approximately 10 mN per microneedle in agar. Meanwhile, the microneedle structure remained intact and no visible tissue damage was observed in the in vivo validation. In porcine models, the SMB-bot achieves site-specific insulin delivery with temporally redistributed exposure, reducing early-phase burst release relative to subcutaneous injection. This platform provides a versatile, clinically translatable strategy for oral biologic delivery, with potential applications in protein/peptide therapy, intestinal disease treatment, and localized systemic drug administration. The current SMB-bot still requires further optimization before clinical translation, including scaling up fabrication for reproducible large-scale manufacturing, further characterization of long-term biodegradation behavior in vivo, optimization of magnetic navigation strategies for deeper intestinal regions, expansion to additional biologic cargoes beyond insulin, comprehensive evaluation of long-term mucosal safety. Future work will focus on scaling fabrication, optimizing long-term biodegradation, and expanding payload types to advance clinical translation.

## Data Availability

The data that support the findings of this study are available from the corresponding author upon reasonable request.
