# Supplementary material for: An Oral Biodegradable Spiny Milli-Ball Robot for Targeted Intestinal Delivery of Biologics with Delayed Onset and Sustained Action
Source: Cyborg Bionic Syst. 2026 Oct 16;7:0716. doi: 10.34133/cbsystems.0716 (PMC13672960; doi:10.34133/cbsystems.0716)
Supplement: Supplementary file 1 [file cbsystems.0716.f1.zip › Supporting Information-CBS (1).docx]

Supporting Information

**An Oral Biodegradable Spiny Milli-Ball Robot for Targeted Intestinal Delivery of Biologics with Delayed Onset and Sustained Action**

*Rong Tan, Xiong Yang, Yajing Shen**

**This PDF file includes:**

Supplementary Text S1. Magnetic Actuation Modeling and Mucosal Penetration Mechanical Model.

Supplementary Fig. S1 Schematic illustration of the fabrication process of the SMB-bot.

Supplementary Fig. S2. Optical images of the SMB-bot and its capsule-compatible configuration.

Supplementary Fig. S3. The pH-responsive dissolution behavior of the SMB-bot in simulated intestinal fluid.

Supplementary Fig. S4. Optical microscopy images of L929 fibroblast cells cultured under control conditions and in the presence of 100 mg SMB-bot extract over different incubation periods.

**Other Supplementary Materials for this manuscript include the following:**

Movie S1. Magnetically Actuated Penetration of the SMB-bot into a Mucosa-Mimicking Agar Substrate

Movie S2. Magnetically guided rolling locomotion of the SMB-bot under high-viscosity mucus conditions

Movie S3. In vivo endoscopic visualization of SMB-bot locomotion and targeted magnetic insertion

**Text S1. Magnetic Actuation Modeling and Mucosal Penetration Mechanical Model.**

The magnetic skeleton of the SMB‑bot is composed of gelatin and Fe₃O₄ nanoparticles, which is equivalent to a uniform magnetic dipole under an external magnetic field.

Magnetic dipole moment:

where M = magnetization of the magnetic skeleton (emu/g), V = effective volume of the magnetic actuation unit (mm³).

Under external magnetic field B, the magnetic torque driving the robot to rotate is:

where θ = angle between the magnetic moment m and magnetic field B. This torque provides the rotational driving force for directional rolling of the SMB‑bot (Fig. 3A).

The magnetic pulling force for translational movement is derived from the magnetic field gradient:

where ∇B = magnetic field gradient (T/m).

The mechanical equilibrium during locomotion is:

 where Ff is: sliding friction force, Fη is viscous resistance of intestinal mucus, mbot is total mass of the SMB‑bot, a is the acceleration of the robot. The linear velocity of the rolling robot is:

where D=6.5 mm (SMB‑bot diameter), ω = angular velocity, I*_rot_* = rotational inertia.

Penetration is driven by magnetic normal force plus gravity component:

 where Fmag,⊥is normal magnetic force, m is the mass of SMB‑bot , g=9.8 m/s2, θ is the inclination angle of the robot relative to the tissue surface. To avoid fracture during insertion:

where N is the number of effective microneedles, Atip is cross‑sectional area of a single microneedle tip, σfail≈37 MPa (measured indentation strength).

**Supplementary Figure**

**Fig. S1. Schematic illustration of the fabrication process of the SMB-bot.** The fabrication procedure begins with drop-casting of the EL100-55 compound solution into the PDMS mould under vacuum-assisted filling, followed by solvent evaporation and drying to form the enteric shell structure. API powders are subsequently filled and compressed into the microneedle cavities, after which HAMA hydrogel is cast and UV-cured to form the backing layer. A PVA binder layer is then introduced to integrate the magnetic actuating skeleton with the microneedle structure.

**Fig. S2. Optical images of the SMB-bot and its capsule-compatible configuration. (A)** Optical image of a single SMB-bot, showing its compact millimeter-scale dimensions suitable for oral administration applications. Insets present enlarged views of the fabricated spiny architecture from different perspectives. **(B)** Optical images showing that three SMB-bots can be compactly packaged within a standard commercial size-00 capsule, demonstrating the excellent capsule compatibility of the system for oral delivery and practical administration. Scale bars, 5 mm.

**Fig. S3. The pH-responsive dissolution behavior of the SMB-bot in simulated intestinal fluid.** The enteric EL100-55 shell gradually dissolved over time under intestinal pH conditions

**Fig. S4. Optical microscopy images of L929 fibroblast cells cultured under control conditions and in the presence of 100 mg SMB-bot extract over different incubation periods.** Cell morphology and adhesion behavior were monitored at 0, 10, and 24 h after incubation. No obvious differences in cell spreading, attachment, or morphology were observed between the control and SMB-bot treatment groups, indicating the good in vitro cytocompatibility of the SMB-bot materials even at a high extract concentration.
